# Kinematic Pattern of the Drag-Flick: a Case Study

**DOI:** 10.2478/v10078-012-0076-7

**Published:** 2012-12-30

**Authors:** María Gómez, Cristina López de Subijana, Raquel Antonio, Enrique Navarro

**Affiliations:** 1 Sport Science Faculty - Technical University of Madrid, Spain.

**Keywords:** drag-flick, biomechanics, shooting, field hockey

## Abstract

The drag-flick is more efficient than hits or pushes when a penalty corner situation is in effect in field hockey. Previous research has studied the biomechanical pattern of the drag-flick, trying to find the cues for an optimal performance. On the other hand, some other studies have examined the most effective visual pick-up of relevant information in shots and goalkeeper anticipation. The aim of this study was to analyse the individual differences in the drag-flick pattern in order to provide relevant information for goalkeepers. One female skilled drag-flicker participated in the study. A VICON optoelectronic system (Oxford Metrics, Oxford, UK) was used to capture the drag-flicks with six cameras. The results showed that the main significant differences between right and left shots (p<0.05) in the stick angles, stick minimum angular velocity and front foot-ball distance were when the front foot heel contacted the floor (T1) and at the minimum velocity of the stick, before the dragging action (T3). The findings showed that the most relevant information might be picked up at the ball-and-stick location before the dragging action.

## Introduction

The penalty corner is one of the most important scoring plays in field hockey (Laird and Sutherland, 2003). This tactical situation appears much more frequently than in other sports like soccer, having a larger impact on the outcome of matches. During the Hockey Champion Trophy 2007, 30% of the 146 goals scored followed penalty corners (Cañal-Bruland et al., 2010). The drag-flick is 1.4 to 2.7 times more efficient than hits or pushes when the penalty corner situation is in effect (McLaughlin, 1997; Piñeiro et al., 2007; Yusoff et al., 2008).

Previous research has studied the biomechanical pattern of the drag-flick, trying to find the cues for an optimal performance (López de Subijana et al., 2010; McLaughlin, 1997; Yusoff et al., 2008). In addition, some research was focused on the goalkeepers’ anticipation when facing a penalty corner (Baker et al., 2009; Cañal-Bruland et al., 2010; Savelsbergh et al., 2002).

An effective visual pick-up of relevant information, apart from an accurate motor execution, is necessary to make a skilled sport performance (Cañal-Bruland et al., 2010). Moreover, in high-speed sports such as hockey, the speed of play and ball velocity dictate that decisions must often be made in advance of the action (Savelsbergh et al., 2002). Therefore, the goalkeeper is required to process visual information and perform in a very limited time frame (Panchuk and Vickers, 2006).

The visual information may be provided by kinematic cues of the opponent’s movement. Goalkeepers may need to pick up the relevant information, such as the direction as well as the height of the shot, from the body kinematics before the penalty-taker hits the ball (Savelsbergh et al., 2002). It is known that skilled performers are more likely to pick up informative anticipatory cues from earlier time points in their opponent’s movement pattern (Baker et al., 2009).

Therefore, not all optical variables provide information equally. Savelsbergh et al. (2002; 2005) showed that body-based cues from the player were more important than those related to the flight of the ball. Other researchers (Williams and Burwitz, 1993) pointed out the importance of the position of the hips, kicking leg and trunk in soccer just before and during contact.

There are some previous studies which showed that goalkeepers who focused more time on the ball-and-stick location saved more penalties than those who followed the ball trajectory after the pusher brought the ball into play (Cañal-Bruland et al., 2010; Panchuk and Vickers, 2006). Panchuk and Vickers (2006) analysed the ocular behaviour of ice hockey goalkeepers while they faced shots taken from different distances. The results showed that 70.53% of the eye fixations were located on the stick-puck (ball) as the shot was prepared and executed. Very few eye fixations were located on body-based cues from the shooter.

Many studies have analysed the ocular and motor behaviour of players when they face a shot in different sports and the cues that they use to predict the direction of the ball (Abernethy, 1990; Müller et al., 2006; Oudejans et al., 1997; Savelsbergh et al., 2005; Starkes et al., 1995; Zawadzki, 2010). To date, none of them have analysed the different drag-flick patterns depending on the direction of the shot, and which were the most useful cues to focus on.

There are many movement variations in the individual technique of each player. Some variations are different movements necessary to adapt to environmental constraints in sport games situations, and others are ‘noise’ (mistakes) of the optimal movement pattern (Beckmann et al., 2010).

Although it is supposed that an expert player may have fewer movement variations and less ‘noise’ than a novice player, there are always variations in the individual technique of each player.

One of the environmental constraints that the player has to face is the position of the goalkeeper during the penalty corner. The player has to make different movements necessary to change the direction of the shot, so it is hypothesized that the player will have different drag-flick patterns depending on the direction of the shot.

The aim of this study was to analyse the individual differences in the drag-flick pattern in order to provide relevant information for goalkeepers.

## Material and Methods

### Participants

One skilled female drag-flicker (20.42 years; 73.6 kg; 171.3 cm; 5 years of experience in drag-flick) participated in the study. This field hockey player was the drag-flicker of the Spanish national team. The participant was requested to provide informed consent prior to participation.

### Measures

The 3D analysis of the drag-flick was performed in the Biomechanics Laboratory of the Faculty of Physical Activity and Sport Sciences at the Technical University of Madrid. A VICON optoelectronic system (Oxford Metrics, Oxford, UK) captured the drag-flicks with six cameras, sampling at 250 Hertz. The experimental space was 5 metres long, 2.5 metres wide and 2 metres high. It was dynamically and statically calibrated with an error of less than 2 centimetres and a static reproducibility of 0.4%. A total of 50 retro-reflective markers (46 body markers and 4 stick markers; 14 mm diameter) were attached to anatomical landmarks following an adapted model from VICON’s kinematics model (Vicon Motion Systems, 2003). The stick markers were placed at the centre of mass position of the stick, at the beginning of the shaft, at the head of the shaft and at the end of the shaft. The player used her own stick approved by the International Hockey Federation (2009). The ball was covered with retro-reflective material to determine its velocity and trajectory. Raw data were filtered using Quintic Spline functions based on Woltring’s CGV method for calculating the smoothing factor (Woltring, 1986).

### Procedures

After a specific warm-up, 15 left trials and 15 right trials at their natural speed were randomly captured from the subject. If the participant did not introduce the ball into the goal area, the trial was rejected. The ball was placed by the subject approximately 1.5 to 2 metres away from the centre of the calibrated area. The drag-flick movement commenced 20 frames before the right foot contacted the floor and continued until 20 frames after the ball release.

**Figure 1:**
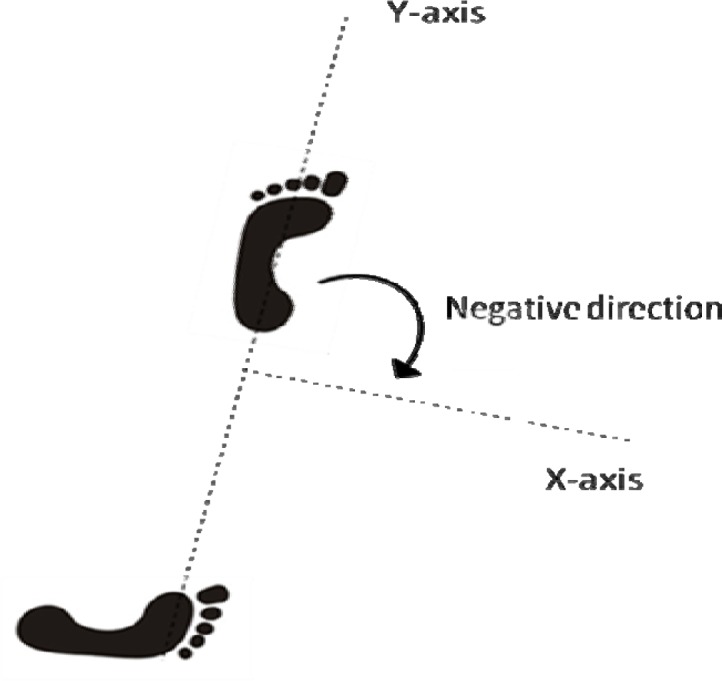
Y-axis and X-axis location in the experimental space of the Biomechanics Laboratory

The ball velocity at release was obtained. The pelvis, upper trunk, and stick angles were calculated considering the line of the double foot contact as the Y-axis, the X-axis 90° from the Y-axis to the right and the Z-axis as the vertical axis. Angular velocities at clockwise were considered as negatives, and those at anticlockwise were considered positives (Figure 1). The angles were computed with the line formed by the upper trunk (shoulder line), pelvis (hip line), and stick with the X-axis on the XY plane. The knee flexion angle was registered for the front leg only. Some kinematic events of the drag-flick were identified, with the corresponding time periods: T1 (front foot heel contact), T2 (maximum angular velocity of the pelvis), T3 (minimum angular velocity of the stick), T4 (maximum angular velocity of the upper trunk), T5 (maximum angular velocity of the stick), T6 (release of the ball) and T7 (maximum velocity of the ball). The event times were normalised considering T1 as 0% and T6 as 100%. The stance width, drag-flick length, front foot-ball distance at T1 and T6, and hip line midpoint-shaft head distance at T1, T3 and T6 were obtained and normalised to the player’s body height.

### Statistical Analysis

Statistical analysis was carried out using SPSS v.15 software (SPSS Inc., Chicago, IL, United States). Means and standard deviations of the study were calculated. Comparison of means between independent groups (right and left trials) was used (U Mann-Whitney). The effect size was calculated using Cliff’s Delta test (Macbeth et al., 2011). The alpha level of significance was set at *p*<0.05 for all statistical tests.

## Results

The ball velocity at release did not differ between the right (22.20 ± 0.80 m/s) and left drag-flicks (22.49 ± 0.68 m/s). During the front heel contact with the floor, as shown in Table 1, the stick position of the right drag-flicks was significantly behind (Z=2.06; *p*<0.05) the stick position of the left ones.

At double foot contact (T1), the distance between the front foot and the ball, and the distance normalised to the player’s body height, were significantly longer (Z=2.34; *p*<0.05) in the right hand side than in the drag-flicks to the left (Table 1).

The minimum angular velocity of the stick (T3) was significantly higher in the right drag-flicks than in the left ones (Z=3.41; *p*<0.001). The angle of the stick in the right shots at T3, as at T1, was significantly greater (Z=3.64; *p*<0.001) than in the left drag-flicks (Table 1).

It was shown in Table 1 that the front foot-ball distance at release (T6) (Z=2.49; *p*<0.01) and the normalised drag-flick length were significantly shorter (Z=2.47; *p*<0.01) in the right drag-flicks than in the left shots.

In the kinematic sequence there were no significant differences between time events at the right and the left shots (Table 2).

## Discussion

The aim of this study was to analyse the individual differences in the drag-flick pattern in order to provide relevant information for goalkeepers. As it was shown in the results the main differences in the drag-flick pattern depending on the direction of the shot occurred before the dragging action of the stick.

Once the goal was scored, the principal criterion to evaluate the efficiency of the drag-flick was the ball velocity. In this study, the drag-flicks shot in both directions (right and left) showed higher velocities (22.20 ± 0.80 m/s right drag-flicks; 22.49 ± 0.68 m/s left drag-flicks) than in the study by López de Subijana et al. (2010) with male hockey players (21.9 ± 1.7 m/s) and female hockey players (17.9 ± 1.7 m/s). These values were also higher than those reported by McLaughlin (1997) (19.1 to 21.9 m/s) and Yusoff et al. (2008) (19.6 to 27.8 m/s). It was noticeable that there were no differences in ball velocities between right and left drag-flicks, so both sides were equally efficient.

Furthermore, there were no significant differences between right and left drag-flick patterns. Both sides showed the same kinematic sequence of peak angular velocities, with the maximum angular velocity of the upper trunk (T4) preceding minimum angular velocity of the stick (T3) (T1-T2-T4-T3-T5-T6-T7 sequence). This kinematic sequence differed from that described by López de Subijana et al. (2010), again with female players, where minimum angular velocity of the stick preceded maximum angular velocity of the upper trunk. The difference between the kinematic sequence of this study and the one described by López de Subijana et al. (2010) could be due to the experience of the players. The participants in the study by López de Subijana et al. (2010) had less experience than the participant in this study. They were not skilled drag-flickers, so their patterns could have been less consistent than the one described in the present study.

Analysing the variables during the kinematic sequence above described, at T1, when the front foot heel made contact with the floor, the distance to the ball (−1.58 ± 0.05 m) and the normalised one (−0.93 ± 0.03 m) were longer in right drag-flicks than the distance to the ball (−1.51 ± 0.07 m) and normalised one (−0.88 ± 0.04 m) in left shots. Shots from both sides showed longer distances than those reported by López de Subijana et al. (2010) (−0.93 to −1.23 m), McLaughlin (1997) (−0.73 to −0.81 m) and Yusoff et al. (2008) (1.01 to 1.66 m). The reason for the longer distances of the right shots at T1 could probably be the position of the stick at that time. The angle between the line of double foot contact (Y-axis) and the stick was higher in right drag-flicks (−90.62 ± 22.96°) than in left hand shots (−77.28 ± 31.80°). The angle of the stick in the right shots was greater than in the left ones, therefore the distance to the front foot was longer in the right drag-flicks than in the left ones. As the time to control the ball was limited, the players chose to prepare with a greater angle in the right shots and to finish with a longer follow-through in the left ones. The difference at the angles showed at T1 was maintained at T3. The stick angle was greater in the right drag-flicks (−96.47 ± 26.50°) than in the left shots (−74.50 ± 33.57°).

At the same time (T3), the results also showed another significant difference, when the minimum angular velocity of the stick was higher in the right drag-flicks (−185.04 ± 31.06°/s) than in the left hand side (−114.75 ± 69.52°/s). The player moved the stick clockwise (‘whipping action’) before the final acceleration (anticlockwise), prior to the ball release, to enhance the dragging action. The whipping action is characterized by minimum angular velocity of the stick. These angular velocities registered in our study were very similar to those reported by López de Subijana et al. (2010) in male (−124.6 ± 112.2°/s) and female hockey players (−194.2 ± 87.5°/s).

Finally, at the moment of the ball release (T6), the distance between the front foot and the ball also differed between the right and left shots. The front foot–ball distance in the right drag-flicks (0.50 ± 0.16 m) and the normalised one (0.29 ± 0.09 m) were shorter than the distance (0.67 ± 0.15 m) and the normalised one (0.39 ± 0.09 m) in the left shots. Moreover, the normalised drag-flick length was also shorter in right shots (1.36 ± 0.19 m) than in left ones (1.63 ± 0.39 m), and both were similar to the male group drag-flick length shown by López de Subijana et al. (2010) (1.38 ± 0.16 m). These differences were opposite to those found at T1 and T3, where the greater distances were at the right drag-flicks. It was considered that when the drag flick was aimed to the left side, the player had to extend the movement because the stick had to cover a longer distance to reach the left side of the goal, since the stick is always at the right hand side of the player.

In accordance with Cañal-Bruland et al. (2010) and Panchuk and Vickers (2006), the results showed that the main differences between right and left drag-flicks were the position of the stick and the ball at the beginning of the shoot.

Although the main limitation of the present study was the number of participants, the data registered from one hockey player allowed us to preview how the right and left drag-flick patterns could differ. Moreover the analysis of the drag-flick was performed in the Biomechanical Laboratory instead of the usual conditions in the field, what could slightly change the normal pattern of the player.

## Conclusion

In summary, the findings of this study showed that the main differences between right and left drag-flicks are the position of the stick and the ball when the front foot heel contacts the floor (T1) and at minimum velocity of the stick, before the dragging action (T3). As the goalkeepers focus on the stick/puck, they should be able to read the orientation of the stick during the dragging action and to anticipate the direction of the shoot.

The differences between right and left drag-flicks shown at T6, the moment of ball release, were not so useful for predicting the direction of the shoot because the goalkeepers might not have enough time to process visual information and perform.

It is remarkable that both side shots were equally efficient because there were no differences in ball velocities between right and left drag-flicks. And, also, that the main events in the kinematic sequence were in the same order.

In conclusion, it is settled the importance for the goalkeepers of focusing on the information before the release occurs, this cue will be more useful than focusing on ball trajectory. Therefore, the goalkeeper should train anticipatory skills using video recording of the players and focusing on the stick and ball position before the release. On the other side the field hockey drag-flicker should practice shooting in selected directions without showing different patterns and trying to avoid the minimal differences in the pattern.

Further research is required to study the right and left pattern of the drag-flick in a larger number of female and male hockey players. In future studies more drag-flick directions could also be included, as the top and bottom of the goal.

## Figures and Tables

**Table 1:** Significant Differences between Right and Left Drag-Flicks (Mean ± Standard Deviations)

	**RIGHT** (M ± SD)	**LEFT** (M ± SD)	**SIZE EFFECT**
**Angular Velocities (°/s)**			
Stick minimum angular velocity (t3) ***	−185.04 ± 31.06	−114.75 ± 69.52	0.78
**Angles (°)**			
Stick angle (t1) *	−90.62 ± 22.96	−77.28 ± 31.80	0.48
Stick angle (t3) ***	−96.47 ± 26.50	−74.50 ± 33.57	0.81
**Distances (m)**			
Front foot - ball distance (t1) *	−1.58 ± 0.05	−1.51 ± 0.07	0.55
Normalised front foot - ball distance (nph) (t1) *	−0.93 ± 0.03	−0.88 ± 0.04	0.55
Front foot - ball distance at release (t6) **	0.50 ± 0.16	0.67 ± 0.15	0.66
Normalised drag-flick length (nph) (t6) **	1.36 ± 0.19	1.63 ± 0.39	0.55

* p<0.05;

** p<0.01 and

*** p<0.001; nph = normalised to player’s height; Size Effect calculated with Cliff’s Delta test

**Table 2:** Event Times and Normalised Event Times (Mean ± Standard Deviations)

	**RIGHT** (M ± SD)	**LEFT** (M ± SD)
**Event times (s)**		

T1	0	0
T2	0.114 ± 0.023	0.120 ± 0.027
T3	0.163 ± 0.017	0.169 ± 0.020
T4	0.158 ± 0.027	0.157 ± 0.026
T5	0.257 ± 0.016	0.261 ± 0.027
T6	0.257 ± 0.013	0.262 ± 0.016
T7	0.264 ± 0.015	0.268 ± 0.016

**Normalised event times (%)**		

T2n	44.26 ± 7.13	45.43 ± 8.14
T3n	63.26 ± 5.03	64.23 ± 4.23
T4n	61.54 ± 9.44	59.70 ± 8.59
T5n	100.04 ± 4.65	99.52 ± 7.27
T6n	100	100
T7n	102.71 ± 3.40	102.35 ± 1.90
